# Clinical communication and caregivers’ satisfaction with child healthcare in Nepal; results from Nepal Health Facility Survey 2015

**DOI:** 10.1186/s12913-018-3857-4

**Published:** 2019-01-08

**Authors:** Charlotte Trimmer, Mats Målqvist

**Affiliations:** International Maternal and Child Health, Department of Women’s and Children’s Health, Uppsala University, SE-751 85 Uppsala, Sweden

**Keywords:** Patient satisfaction, Patient communication, Child health, Nepal

## Abstract

**Background:**

Patient satisfaction is an important measure of quality of care and a determinant of health service utilisation and the choice of health facility. Measuring patients’ experiences is important for understanding and improving the quality of care at health facilities. The aim of this study was to assess levels and identify associated factors of caregivers’ satisfaction and provider-caregiver communication within child healthcare in Nepal.

**Methods:**

Secondary analysis of Sick Child Exit Interviews (*n* = 2092) sourced from 2015 Nepal Health Facility Survey data. Satisfaction was measured through caregivers’ satisfaction with services received and their willingness to recommend the health facility visited. Communication indicators were chosen based on the 2014 WHO IMCI guidelines and aggregate communication scores were calculated based on the number of indicators acknowledged during assessments. Logistic regression was used for analysis.

**Results:**

Although most respondents (82.1%) reportedly were satisfied with the care provided, only 35.9% experienced good communication with their providers. Caregivers who had ever attended school were more likely to be satisfied with services (1.44, CI 95% 1.04–1.99). Type of provider, sex of child or who the caregiver was had no association with caregivers’ satisfaction. Having been given a diagnosis doubled the chances of satisfaction (AOR 2.04, 95% CI 1.38–3.00), as did discussion of the child’s growth (OR 1.71, 95% CI 1.06–2.76) and having discussed any of the included topics (AOR 1.98, CI 95% 1.14–3.45).

**Conclusions:**

Interventions to improve healthcare staff’s communication skills are needed in Nepal to further enhance satisfaction with services and increase quality of care. However, this is an area that need further investigation given the high levels of satisfaction displayed despite poor communication. Other factors in the health care exchange between provider and clients are influencing the level of satisfaction and need to be identified and promoted further. High-quality care is no longer a goal for the future or only for high income settings; it is essential for reaching global health goals.

## Background

Utilization of health services has increased in many low-income and middle-income countries (LMICs] over the past decades through a determined focus on increasing access [1]. Nevertheless, we know little about the quality of care received in this context. Quality of care is not only about the medical quality of clinical care, but also includes patient experience [2]. Medical quality applies to the use of clinical knowledge and method to solve a health problem, whereas patient experience relates to the responsiveness of the health system to fulfil non-health needs and meet expectations. Patient experience is intrinsically linked to the clinical care received and also associated with better health outcomes [3, 4], as satisfaction has a strong relationship to patients’ adherence to medication and other interventions. Patient satisfaction is thus an important factor to ensure quality of care, and it also influences the sustainability and endurance of services. [5, 6]. Furthermore, information on patient satisfaction can assist in identifying system problems like gaps in coordination and communication.

An essential component of patient experience is communication between provider and patient. Effective clinical communication is linked to health care providers’ capability to understand their patient’s needs and level of understanding, and adjust to it [7]. Improved communication between provider and practitioner has been shown to result in health outcomes [8]. Thus, patient-centred approaches, based on cooperation and compromise, are slowly replacing the more traditional “paternalistic model” of provider-patient relationships [9]. Patients who take an active role in provider-patient interactions also tend to make better decisions about their own care, as they are more likely to ask questions and have a better understanding of medical explanations [4, 10].

Despite the growing evidence concerning the influence of provider-patient communication on health outcomes and caregiver behaviours in high-income settings, the same is not always the care in LMIC settings, where the quality and impact of provider-patient communication is not so well documented. However, it has been identified as sub-optimal in a variety of settings [11, 12]. It is important to study these relationships and their effects in different contexts; as health systems, attitudes towards healthcare, discriminations, and traditional beliefs all play a role in perceived quality of healthcare. Relationships found in one context or environment may not be found in another. Only a few studies have been performed on patient satisfaction and provider-patient communication in the South Asia. A 2008 study on the expectations of Nepalese patients on provider–patient communications found patients expressing a strong preference for patient-centred communication, although they were not very concerned with the sharing of power and control [13]. More evidence is needed of the link between good communication and improved health outcomes for patients.

Communication is especially important when talking about child health, as the provider and the patient’s caregiver are both reliant on each other to provide information and care to aid a child, who cannot communicate themselves.

Understanding the determinants and outcomes of patient communication is essential in LMICs such as Nepal, which bear a disproportionate burden of childhood morbidity and mortality when compared to higher income settings. Therefore, the aim of this study was to assess levels and identify associated factors of caregivers’ satisfaction and provider-caregiver communication within child healthcare in Nepal.

## Methods

This quantitative cross-sectional study sources data from the Service Provision Assessment (SPA] surveys, which are conducted by the Demographic and Health Survey Programme (DHS). The most recent SPA survey addressing Nepal was the 2015 Nepal Health Facility Survey (NHFS) [14]. This analysis focuses on one-time data from exit interviews with caregivers for visits for sick children. Hereafter, when referring to patient satisfaction or provider-patient communication it should be noted that what is recorded is the caregivers’ perspective, even if it is the sick child in their care that is the actual patient. SPA surveys include assessment of facility inputs and processes and interviews with the caretakers of sick children about information relayed to them about their children from the health service provider.

### Study setting

The 2015 NHFS collected information from health facilities run by government, private not for profit Non-Governmental Organisations (NGOs), private for profit and missionary organisations across all 75 districts of Nepal. The sample of health facilities was representative of the three main ecological regions of the country and the six development regions with stratification achieved by separating the facilities by type within each of the 13 assigned development-ecological areas. [14].

### Study population

The sample health facilities for the 2015 SPA were selected by randomly selecting 1000 facilities from a list of all the country’s facilities stratified by type (e.g. hospitals and health centres) and managing authority (e.g. government or private). At each of the 1000 facilities, sample patients were selected using systematic random sampling.

The NHFS defined health service providers as personnel providing consultation services, counselling, health education or laboratory services. Health workers were not counted as providers if they only took measurements or completed registers and did not provide professional client services. The NHFS only sampled providers who were present at the facility and who provided relevant services [15].

For child health consultations, only children younger than age 5 presenting with an illness (rather than an injury or a skin or eye infection exclusively) were selected for observation [15]. The caregivers of such children were identified and systematically selected based on their presence at the time of the survey visit. The interviewers attempted to conduct exit interviews with all primary caregivers before they left the facility.

### Data collection

The 2015 NHFS questionnaires were based on generic questionnaires from the MEASURE Demographic and Health Surveys (DHS) project [15], which were modified to fit the Nepal context and translated into Nepali. Computer-assisted personal interviewing (CAPI) and computer-assisted field editing (CAFE) programs were developed in English and Nepali while the questionnaires were being translated.

The 2015 NHFS’s interviewers observed consultations with under 5-year old sick children by recording the information shared between caregivers and providers and the processes providers followed when assessing patients, carrying out procedures, and providing treatment. The observation protocols the interviewers followed were the primary source of the data to assess whether or not consultations had acceptable standards of content and quality [14].

Following each consultation, caregivers participated in exit interviews to elicit their perceptions of information and services received. In addition, providers were interviewed and asked detailed questions about their in-service training and the supervision they received, as these factors influence the quality of services provided and communication with caregivers and level of caregiver satisfaction with the service delivery environment.

### Variables

Patient satisfaction was measured against caregivers’ opinions of services provided on the day in question and whether or not they would recommend friends and family to use the facility (yes or no). Caregivers’ opinions were scored against the Likert scale of very satisfied (1), fairly satisfied (2), neutral (3), fairly dissatisfied (4) and very dissatisfied (5) [16]. The scale was reduced to the two categories of ‘satisfied’ (including very satisfied and fairly satisfied) and ‘not satisfied’ (including neutral, fairly dissatisfied and very dissatisfied). The two patient satisfaction variables were then combined into a single overall patient satisfaction indicator.

Provider-patient communication was defined through six indicators of quality communication from the SPA dataset: (1) Provider told the caregiver the child’s illness. (2) Provider told the caregiver the symptoms that would indicate a need for immediate return to the facility, (3) Provider scheduled or discussed a non-emergency return visit, (4) Provider counselled the caretaker on feeding the child, (5) Provider discussed the sick child’s growth, and (6) Provider discussed general nutrition. An aggregate score combining all six quality communication indicators was then formed. This rated each respondent’s experience of communication out of six, with six being the highest, and indicating that all six indicator topics were communicated. This score was then made into an ‘Overall Communication Score’, this grouped the scores into ‘Good Communication’, which responded to the provider discussing three or more of the topics and ‘Poor Communication’, which responded to the provider discussing none to two of the topics. Indicators were assessed based on the caretakers’ recall of this communication immediately following the clinical consultation.

This study did not consider data on the communication of specific conditions and medication for child patients as such data could be related to the quality of technical health care delivery rather than patients’ experiences. These variables were also excluded to increase the confidentiality and generalisability of findings.

Potential confounders and covariates of provider-patient communication and of patient satisfaction were identified from recent literature. At the patient level, predictors included the caregiver and child’s sociodemographic characteristics, as well as caregiver’s actions before the visit, for example, the patient’s healthcare payment plan. At the provider level, providers’ position (doctor or clinical officer, nurse etc.), and sex were included in the study. As these variables have been shown to influence patient satisfaction and health outcomes in previous studies [12, 17].

### Statistical analyses

Data was analysed using version 25 of the *Statistical Package for the Social Sciences* (SPSS). Due to the cluster sampling of the population, it was important to weight adjust the sample in order to improve the representability and generalisability of the findings. Context-specific weighting was provided within the dataset. The percentages discussed throughout this study, are weighted percentages. Data was analysed using Pearson’s Chi Squared and binary logistic regression analysis.

### Ethical considerations

Permission to use the data for this study was granted from the DHS. As this was an observational study based on secondary data, there were minimal risks associated with participation in this study.

Questionnaires used for the purposes of the study were relevant to the health issues and context of the Nepali population. It was adapted from previous surveys and approved by various government stakeholders, ministries, agencies, NGOs and donors.

NFHS personnel acquired informed consent from facility in-charges and individual respondents prior to data collection and interviews. Respondents were told they could ask questions or end interviews at any time. The identity of respondents and providers was anonymised after data collection.

## Results

After excluding duplicates and missing responses a total of 2092 cases were included in the study’s dataset. The majority of caregivers who took part in the exit interviews were the mothers of the child patients (77.7%) with 62.8% of the caregiver respondents had some experience of schooling. The most common ethnicity among respondents was Brahman or Chhetri (30.5%). Most of respondents were under 46 years of age (92.9%) and 55.4% of child patients were boys and 44.6% girls (Table 1). Fifty-one per cent of participating health care providers were health assistants, (55.4% male). Student or trainee providers were only involved with the sick child assessment in 13% of cases. Around 40% of respondents got to see their providers immediately (Table 1).

**Table 1:** Provider and patient characteristics (*n* = 2092)

Provider characteristics	n	%	Weighted %
Provider Category			
Nurse	183	8.2	12.4
General Medical Doctor	25	1.1	1.0
Specialist Medical Doctor	316	14.2	14.6
Medical Officer	567	25.4	15.8
Health Assistant	1129	50.7	55.9
Other	9	0.4	0.4
Sex of Provider			
Female	491	22.0	25.7
Male	1738	78.0	55.4
Student Providers Involved			
Yes	349	15.7	13.0
No	1874	84.3	87.0
Waited more than 20 min to see provider			
Yes	1063	47.7	46.7
No	1166	52.3	53.3
Patient characteristics			
Sex of Child			
Female	963	43.2	44.6
Male	1266	56.8	55.4
Respondent’s Relationship to Child			
Mother	1797	82.2	77.7
Father	203	9.1	9.0
Aunt/Uncle	42	1.9	2.8
Grandparent	111	5.1	7.7
Other	33	1.5	2.7
Ever Attended School			
Yes	1520	69.5	62.8
No	666	30.5	37.2
Respondent’s Caste/Ethnicity			
Brahaman/Chhetri^a^	795	36.4	30.5
Newar^a^	89	4.1	3.5
Janajati	508	23.2	24.0
Muslim	89	4.1	5.8
Dalitis	318	14.5	14.1
Terai/Madhesi	369	16.9	21.5
Other	18	0.8	0.7

^a^Advantaged caste/ethnic group

Most caregiver respondents were satisfied with the care received by their children (82.1%) in spite of only about 40% of respondents getting to see a provider immediately (Table 1). They were more likely to have been satisfied if they had been attended by a medical doctor (OR 1.39, CI 95% 1.04–1.86) or if the caregiver had ever attended school (OR 1.53, CI 95% 1.12–2.10). Schooling had a positive impact on patient satisfaction even after adjusting for type of provider (1.44, CI 95% 1.04–1.99) (Table 2).

**Table 2:** Association between Patient satisfaction/Provider-patient communication (aggregate scores) and provider and patient characteristics in Nepal. Univariate logistic regression displaying crude odds ratios

	Patient satisfaction			Provider-patient communication		
Provider characteristics and health system factors	Satisfied (weighted %)	Not satisfied (weighted %)	OR (95% CI)	Good communication (weighted %)	Poor communication (weighted %)	OR (95% CI)
Medical doctor						
*Yes*	85.3	14.7	**1.39 (1.04–1.86)**	43.2	56.8	**1.57 (1.24–1.99)**
*No*	80.6	19.4	Ref	32.6	67.4	Ref
Nurse						
*Yes*	82.1	17.9	1.00 (0.60–1.66)	30.0	70.0	0.74 (0.50–1.09)
*No*	82.1	17.9	Ref	36.8	63.2	Ref
Health assistant						
*Yes*	80.3	19.7	0.76 (0.56–1.02)	33.3	66.7	0.78 (0.61–1.00)
*No*	84.3	15.7	Ref	39.1	60.9	Ref
Male provider						
*Yes*	82.1	17.9	1.00 (0.71–1.43)	35.7	64.3	0.96 (0.73–1.26)
*No*	82.0	18.0	Ref	36.7	63.3	Ref
Waiting time more than 20 min						
*Yes*	83.5	16.5	1.21 (0.89–1.64)	37.5	62.5	1.14 (0.90–1.45)
*No*	80.8	19.2	Ref	34.5	65.5	Ref
Patient characteristics						
Accompanied by mother						
*Yes*	84.9	15.1	1.29 (0.87–1.93)	37.9	62.1	**1.50 (1.08–2.09)**
*No*	81.3	18.7	Ref	28.9	71.1	Ref
Male child						
*Yes*	81.1	18.9	0.86 (0.64–1.17)	35.7	64.3	0.98 (0.77–1.24)
*No*	83.3	16.7	Ref	36.2	63.8	Ref
Ever attended school						
*Yes*	84.5	15.5	**1.53 (1.12–2.10)**	40.7	59.3	**1.79 (1.36–2.36)**
*No*	78.0	22.0	Ref	27.7	72.3	Ref
Belonging to advantages case/ethnicity						
*Yes*	83.6	16.4	1.18 (0.87–1.59)	42.5	57.5	**1.53 (1.21–1.94)**
*No*	81.3	18.7	Ref	32.6	67.4	Ref

Despite demonstrating high levels of satisfaction, only over a third (35.9%) of respondents reported receiving good communication from their provider. Having been attended by a medical doctor increased the chances of receiving good communication (having discussed three or more of the topics included in the survey) (OR 1.57, CI 95% 1.24–1.99). Sex of the child or the provider was not related to level of satisfaction or communication; but if the care giver was the mother, and if the care giver had attended school or belonged to an advantaged caste/ethnic group increased chances of receiving better communication during provider-patient interaction (Table 2). The multivariate logistic regression analysis of these results showed that the association between good communication and provider type, mother as caregiver and caste/ethnicity was confounded by the level of education of the caregiver, which was the only determinant that displayed an association with provider-patient communication (AOR 1.55, CI 95% 1.16–2.08).

In the current study, most respondents were seen to have discussed one or two of the topics included in the survey (28.0 and 28.2% respectively). Having discussed or carried out any of the six topics doubled the chances of a patient being satisfied (AOR 1.98, CI 95% 1.14–3.45, adjusted for caregiver’s education) (Table 3). Most important among the six topics were being given a diagnosis and to have discussing the child’s growth, both being associated with increased patient satisfaction. In most cases (84.3%), the provider had told the caregiver the diagnosis of the child. However, in about half of the assessments the provider did not discuss the other five topics. General nutrition was discussed the least, at 9.3% of the time (Table 3).

**Table 3:** Frequencies and weighted percentages of provider-patient communication in Nepal 2015 (*n* = 2092). Multivariate logistic regression displaying odds ratios adjusted for caregiver’s education (ever attending school)

Variable			Overall patient satisfaction			
			Satisfied	Not satisfied		
	n	%	%	%	AOR	CI 95%
Provider gave diagnosis						
*Yes*	1867	84.3	86.4	74.6	**2.04**	**1.38–3.00**
*No*	317	15.7	13.6	25.4	Ref	
Provider discussed signs/symptoms for immediate return						
*Yes*	1053	45.6	46.8	40.3	1.22	0.89–1.67
*No*	1121	54.4	53.2	59.7	Ref	
Provider discussed reasons for follow-up						
*Yes*	1017	44.7	45.9	39.3	1.26	0.92–1.71
*No*	1185	55.3	54.1	60.7	Ref	
Provider discussed growth						
*Yes*	294	14.7	15.8	9.7	**1.71**	**1.06–2.76**
*No*	1892	85.3	84.2	90.3	Ref	
Provider discussed normal feeding practices						
*Yes*	318	12.8	13.4	9.6	1.38	0.85–2.26
*No*	1846	87.2	86.6	90.4	Ref	
Provider discussed general nutrition						
*Yes*	231	9.3	10.0	6.3	1.57	0.86–2.86
*No*	1943	90.7	90.0	93.7	Ref	
Provider discussed any of the above topics						
*Yes*	129	7.9	82.2	17.2	**1.98**	**1.14–3.45**
*No*	1961	92.1	68.9	31.1	Ref	
Provider discussed three or more of above topics (Good communication)						
*Yes*	775	35.9	37.3	30.2	1.30	0.94–1.81
*No*	1315	64.1	62.7	69.8	Ref	

## Discussion

The results show a high level of satisfaction of caregivers with the health care that their under 5 year olds receive, with 82% of respondents being satisfied with their care. This was a higher percentage than compared to other studies. In Ethiopia, under two-thirds of respondents reported being satisfied with their healthcare provider [17], and around 50% in another Africa-based study [18]. In the Korean setting, the level of satisfaction was higher, 75% [19], suggesting that cultural context may play a large part in the results of this indicator.

The increased likelihood of patient satisfaction when receiving good communication shows on the importance of the relationship between provider and patient for the quality of care. Good communication has been linked to patient satisfaction in the shorter term [12] as well as to improved health outcomes and patient adherence in the longer term [20, 21]. Good communication was seen in over a third of respondents, corresponding to a similar result in a study conducted across seven African countries [12].

Providers were seen to describe the sick child’s diagnosis in the majority of cases (84.3%) and discussed other indicator topics in less than half of cases. When looking at the IMCI guidelines, discussion of feeding practices and nutrition feature prominently, however in this study these discussions happened in less than 15% of cases. This result is higher than in a similar study conducted in seven African countries, which had a mean of 54%, with only 10% reporting on counselling of feeding practices [12]. To be given the diagnosis was seen to be highly associated with patient satisfaction, corresponding with findings in Ethiopia, linking high patient satisfaction with provision of diagnosis [17]. It also relates to other studies which link providing information and patient involvement in decision-making with overall satisfaction and willingness to return [22].

Seeing a medical doctor was found to lead to an increased likelihood of experiencing good communication whilst being seen by a health assistant was found to decrease the likelihood of good communication. Medical doctors and health assistants have very different levels of education and training, yet this does not necessarily mean that this would be the only factor that influence their communication. Misunderstandings about information delivery between health care professionals, may also be an underlying reason for an inequality in patients’ communication. Health assistants may be unsure what information has been given to the patients previously or by another healthcare professional, which may have hampered the quality of the information delivered, as described in a French study looking into medical information received from providers and patient satisfaction [23].

These findings correspond with other study findings, highlighting the importance of assessing provider communication, as well as technical care and health outcomes, when gauging the impact of task-shifting responsibilities from doctors and nurses to staff with less training [12]. This is especially relevant in Nepal where local female community health volunteers (FCHVs) play a major role in promoting the health of mothers and children. FCHVs also promote the use of health services, raise awareness on health issues and are involved in drug distribution and disease management [24]. In 2009, Nepal’s FCHV programme won the Global Alliance for Vaccines and Immunisation (GAVI) award for the highest average annual rate of reduction of child mortality among all 72 GAVI-supported countries [25]. Clearly good provider-patient communication should be encouraged by all levels of health care providers including FCHVs to improve health outcomes.

Nepali society has a unique element that may influence healthcare provision and satisfaction, its caste and ethnicity hierarchy. There is much debate over the exact influence it has, but in the current study the advantageous group of respondents were seen to have a marked increased likelihood of receiving good communication, in comparison to those not from the advantageous group, which may indicate discrimination from providers. Providers such as medical doctors and nurses are often from advantageous caste/ethnic backgrounds [13], which may impact patients not from these backgrounds, as seen in other studies which found a link between ethnic backgrounds and communication in healthcare [26].

Furthermore, results indicate that there is an association between respondent education and provider communication. Educated caregivers may be more likely to start and maintain communication with providers or may have a higher capacity to understand providers and remember what has been communicated. Those with more education might also not have the same levels of perceived stigma against them from health care professionals, therefore they would be more comfortable in communicating.

Much of the emphasis in patient satisfaction surveys has been on measuring health care use rather than quality. Policymakers need to decide how to measure the quality of health care using robust, consistent, and financially efficient tools and metrics. Countries and cooperating NGOs should then rigorously test solutions for improving quality to guarantee that interventions and policies are appropriate [12, 27]. The World Bank’s service delivery indicator surveys are designed to collect clinical performance and efficiency data, easily and rapidly, and are promising for global data collection on the quality of care [27].

Most empirical studies on the effect of provider-patient relationships on health outcomes have been observational and have therefore not assessed causality. As observational bias may influence study results, efforts have been made to reduce such bias by making discrete observations. There is also the danger of response bias where respondents answer questions in ways they think will be deemed favourable by others such as interviewers or researchers. The risk of this was high for NHFS data as the interviews were administered through direct questions. However, the NHFS’s assurance of respondents’ anonymity and that the purpose was to assess facilities and providers rather than respondents themselves would have helped to decrease such bias. The high levels of satisfaction indicated by the caregivers may have, however, been affected by their fear to brand their interactions with providers negatively, especially if they envisaged seeking further treatment or consultations from them. There will also have been the risk of respondents’ desires to finish questionnaires quickly due to tiredness or hunger.

Importantly, information was not available on the structural characteristics of the health facilities and the individual characteristics of physicians and patients. This was due to the lack of questions on facility variables in the NHFS sick child exit interviews. Further studies could combine their analysis with other sections of the NHFS.

Among the different types of health facilities, hospitals were oversampled in the NHFS and in most SPA surveys. This has been found to be an influencing factor on patient satisfaction and communication, especially in lower income settings [12, 18]. However, due to the stratified, cluster nature of the sampling and the weighting of the dataset before analysis, the findings can be applied to Nepal’s population as a whole when looking at the provider and respondent level characteristics.

## Conclusions

The results highlight the importance of testing strategies and interventions that enhance clinical inter-personal communication as a means of improving health outcomes and patient experiences in LMICs. Countries that promote patient-centred health services need to carry out more in-depth research on the determinants of patient satisfaction in their respective cultures. High-quality care is no longer a goal only for the distant future in countries such as Nepal; it is essential for reaching the global health goals and should be an elemental responsibility of every health system.

## Abbreviations

AOR: Adjusted odds ratio
CAFE: Computer-assisted field editing
CAPI: Computer-assisted personal interviewing
CI: Confidence interval
FCHV: Female community health volunteer
GAVI: Global alliance for vaccines and immunisation
IMCI: Integrated management of childhood illness
LMIC: Low and middle income country
NGO: Non-governemental organization
NHFS: Nepal health facility survey
NHSS: Nepal health sector strategy
SPA: Service provision assessment
